# α-Actinin and Filamin Cooperatively Enhance the Stiffness of Actin Filament Networks

**DOI:** 10.1371/journal.pone.0004411

**Published:** 2009-02-09

**Authors:** Osigwe Esue, Yiider Tseng, Denis Wirtz

**Affiliations:** 1 Department of Chemical and Biomolecular Engineering, Johns Hopkins University, Baltimore, Maryland, United States of America; 2 Department of Early Stage Pharmaceutical Development, Genentech, Inc., South San Francisco, California, United States of America; 3 Department of Chemical Engineering, University of Florida, Gainesville, Florida, United States of America; University of Birmingham, United Kingdom

## Abstract

**Background:**

The close subcellular proximity of different actin filament crosslinking proteins suggests that these proteins may cooperate to organize F-actin structures to drive complex cellular functions during cell adhesion, motility and division. Here we hypothesize that α-actinin and filamin, two major F-actin crosslinking proteins that are both present in the lamella of adherent cells, display synergistic mechanical functions.

**Methodology/Principal Findings:**

Using quantitative rheology, we find that combining α-actinin and filamin is much more effective at producing elastic, solid-like actin filament networks than α-actinin and filamin separately. Moreover, F-actin networks assembled in the presence of α-actinin and filamin strain-harden more readily than networks in the presence of either α-actinin or filamin.

**Significance:**

These results suggest that cells combine auxiliary proteins with similar ability to crosslink filaments to generate stiff cytoskeletal structures, which are required for the production of internal propulsive forces for cell migration, and that these proteins do not have redundant mechanical functions.

## Introduction

Actin monomers in the presence of crosslinking or bundling proteins assemble into filamentous networks that are significantly stiffer than F-actin networks in the absence of crosslinking and bundling proteins [1]–[3]. These earlier reports mostly characterized actin filament assembly, gelation kinetics, and ensuing changes in their mechanical properties induced by a single F-actin crosslinker. However, in cells, these auxiliary proteins often localize in the same subcellular areas. The close spatial proximity of different crosslinking proteins suggests that these proteins may cooperate to organize F-actin stuctures to drive complex cellular functions during cell adhesion, motility and division. Here we hypothesize that α-actinin and filamin, two major F-actin crosslinking proteins that are both present in the lamella of adherent cells [4]–[8], display synergistic mechanical functions.

Filamin and α-actinin are critical to structural functions of skeletal and smooth muscle cells [6], [7], [9]. Both α-actinin and filamin are involved in cell signaling by connecting integrins to the cytoskeleton [10], [11]. There is no evidence that filamin and α-actinin interact directly, but they simultaneously interact with F-actin, with similar association and dissociation rates, at different actin binding regions with little evidence of competition [12], while other auxiliary proteins such as tropomyosin [13] and talin [10] compete for these sites. FATZ and myozenin are Z-line proteins that each individually both bind and form complexes with both α-actinin and filamin in skeletal muscles to help promote F-actin function and regulate cytoskeletal arrangements [9], [14]. Both α-actinin and filamin have two actin-binding sites separated by a relatively flexible molecular arm. Therefore, α-actinin and filamin mediate the formation of orthogonal actin filament networks at low concentration [3], [15]–[20]. At high concentrations, they induce the formation of bundles above a crosslinking-to-bundling threshold concentration, which are relatively disorganized compared to F-actin bundles formed by *bona fide* F-actin bundling protein fascin [19].

Our previous work has shown that F-actin bundling protein fascin and F-actin crosslinking protein α-actinin can work together to enhance the mechanical properties of F-actin networks more efficiently than these proteins alone [21], [22]. Here we use quantitative rheology to investigate whether combining two *bona fide* crosslinking proteins, α-actinin and filamin, may affect the mechanical properties and dynamics of networks differently than α-actinin and filamin alone.

## Results

### α-actinin and filamin synergistically enhance the stiffness of F-actin networks

**Figure 1:**
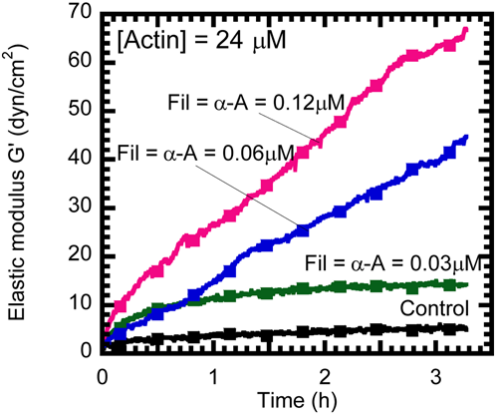
Gelation kinetics of actin filament networks in the presence of equimolar concentrations of F-actin crosslinking proteins α-actinin, and filamin. Time-dependent elastic modulus is measured using a strain-controlled rheometer. The imposed deformation amplitude to measure the elastic modulus was 1% and the shear frequency was 1 rad/s. The concentration of actin was 24 μM.

We monitored the gelation of actin solutions in the presence of either α-actinin or filamin or both using a cone-and-plate rheometer. A strain-controlled rheometer measures the elastic modulus, *G'* (defined as the propensity of the polymers to rebound after shear deformation), and the viscous modulus, *G”* (defined by how much the specimen can flow under stress), of the protein solutions following the onset of actin filament assembly. Upon addition of polymerizing salt, solutions of monomeric actin rapidly formed filamentous networks that exhibited an elasticity of ∼6 dynes/cm^2^ in the absence of crosslinking proteins (Fig. 1).

**Figure 2:**
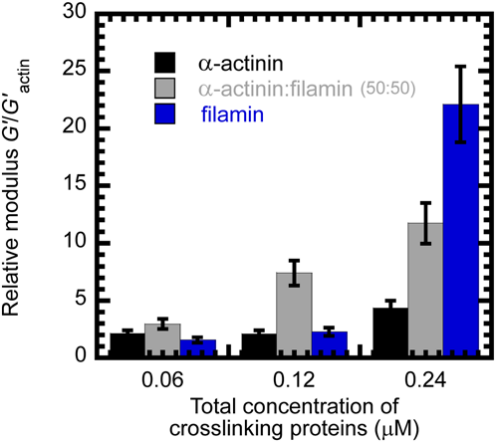
Elastic modulus of F-actin networks in the presence of α-actinin, filamin or both. Steady state elastic modulus of F-actin networks in the presence of α-actinin only (black columns), both filamin and α-actinin (50:50 molar ratio; grey columns), or filamin only (blue columns). was measured using a strain-controlled rheometer. The total concentration of F-actin crosslinking proteins is indicated. The imposed deformation amplitude to measure the elastic modulus was 1% and the shear frequency was 1 rad/s. The concentration of actin was 24 μM.

In the presence of equimolar concentrations of α-actinin and filamin at 0.03 μM, 0.06 μM, and 0.12 μM each, the actin filament network elasticity increased in 3 h to 15 dynes/cm^2^, 45 dynes/cm^2^ and 68 dynes/cm^2^, respectively (Fig. 1). It is no surprise that the network elasticity increased with increasing concentration of crosslinking proteins α-actinin and filamin (Fig. 1). However for the same total molar ratio of actin to auxiliary protein, the effect of combining α-actinin and filamin on F-actin network elasticity was much stronger than the separate effects of either α-actinin or filamin (Fig. 2). The elastic modulus of the networks normalized to the network modulus of F-actin alone was plotted as a function of total auxiliary protein concentration (Fig. 2). At a concentration of 0.12 μM, α-actinin and filamin alone increased the elasticity of F-actin by 2-fold. In contrast, combining 0.06 μM α-actinin with 0.06 μM filamin resulted in a 8-fold increase in network elasticity (Fig. 2). For higher auxiliary protein concentrations, this effect was qualitatively similar, but quantitatively different. Combining 0.12 μM of both α-actinin and filamin increased the elasticity ∼12-fold while adding 0.24 μM α-actinin alone only resulted in a 4-fold increase (Fig. 2). Solutions containing 0.24 μM filamin is above the crosslinking-to-bundling threshold of filamin in F-actin solutions and, therefore, resulted in an elasticity that was ∼22-fold higher than that of F-actin alone (Fig. 2).

### Combining α-actinin and filamin drastically reduce the mobility of actin filaments in networks

**Figure 3:**
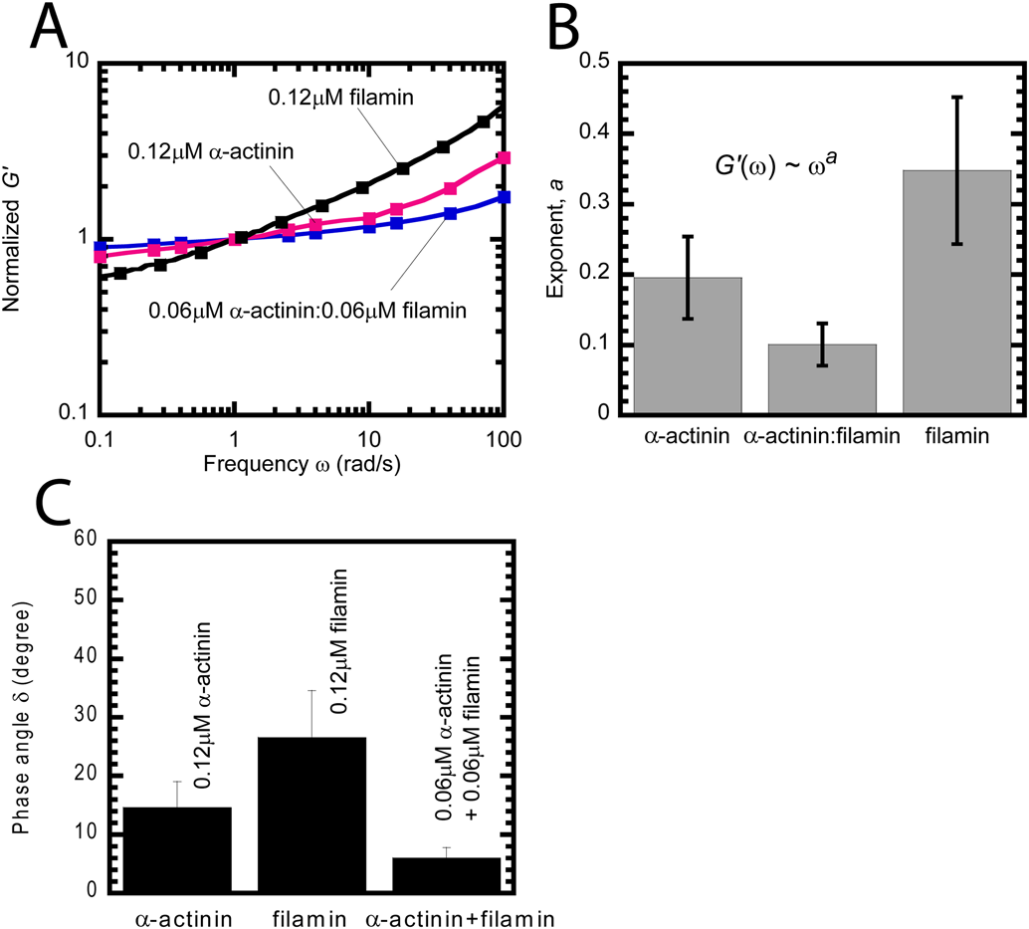
Viscoelastic properties of F-actin networks in the presence of α-actinin, filamin or both. A. Frequency-dependent elastic modulus of F-actin networks in the presence of either 0.12 μM α-actinin, 0.12 μM filamin, or 0.06 μM α-actinin+0.06 μM filamin. The amplitude of the deformation was 1%. Elastic moduli are normalized by their value at 1 rad/s. B. Slope *a* of the elastic modulus obtained from a power-law fit of *G'*(ω)∼ω*^a^* shown in panel A. C. Phase angle of F-actin networks, which compares the viscous modulus *G”* to the elastic modulus, *G'* as δ = tan^−1^(*G”*/*G'*). The phase angle of water and F-actin without crosslinking proteins is 90° and 30°, respectively.

After the elastic modulus reached a steady state value, we measured the rheological response of F-actin networks to oscillatory shear deformations of small amplitude and increasing frequency ω. Such measurements probe the ability of filaments to move and, therefore, relax mechanical stresses inside the networks [23]. The elasticity, *G'*(ω), of F-actin networks increased with frequency in the presence and absence of auxiliary proteins (Fig. 3A). The slope of this increase is dependent on the ability of filaments to move and relax mechanical stresses within the network. Filament movement can be restricted by entanglements formed by topologically overlapping filaments and/or by the crosslinking activity between filaments, which can also impede filament movements.

The elasticity of F-actin networks containing 0.12 μM of either α-actinin or filamin increased steadily with frequency (Fig. 3A). However, the elasticity of F-actin containing both α-actinin and filamin was significantly less dependent on frequency (Fig. 3A). This result indicates that actin filaments inside networks containing both α-actinin and filamin are less mobile and, therefore, less inclined to relax mechanical stresses due to enhanced interfilament interactions than in networks containing only α-actinin or filamin. The frequency-dependent elasticity profiles (Fig. 3A) were fit to power laws of frequency, *G'*(ω)∼ω*^a^*, with an exponent, *a*, that describes the steepness of the frequency dependence of *G'*(ω). The exponent, *a*, for networks of F-actin containing both α-actinin and filamin was significantly lower than for networks containing either α-actinin or filamin alone (Fig. 3B). This result suggests that actin filaments in networks containing both α-actinin and filamin can slide less readily and, therefore, relax mechanical stresses than actin filaments in networks containing only α-actinin or filamin.

Moreover, the phase angle, δ = tan^−1^(*G”*/*G'*), which compares the elastic and viscous moduli of these networks was lowest for networks containing both α-actinin and filamin (Fig. 3C). By comparison, the average phase angle of a glycerol solution and a 24 μM F-actin network is 90° and 30°, respectively. This result indicates that F-actin networks containing α-actinin and filamin are, from a rheological point of view, more solid-like than networks containing only α-actinin or filamin.

### F-actin networks are mechanically more resilient in the presence of both α-actinin and filamin

**Figure 4:**
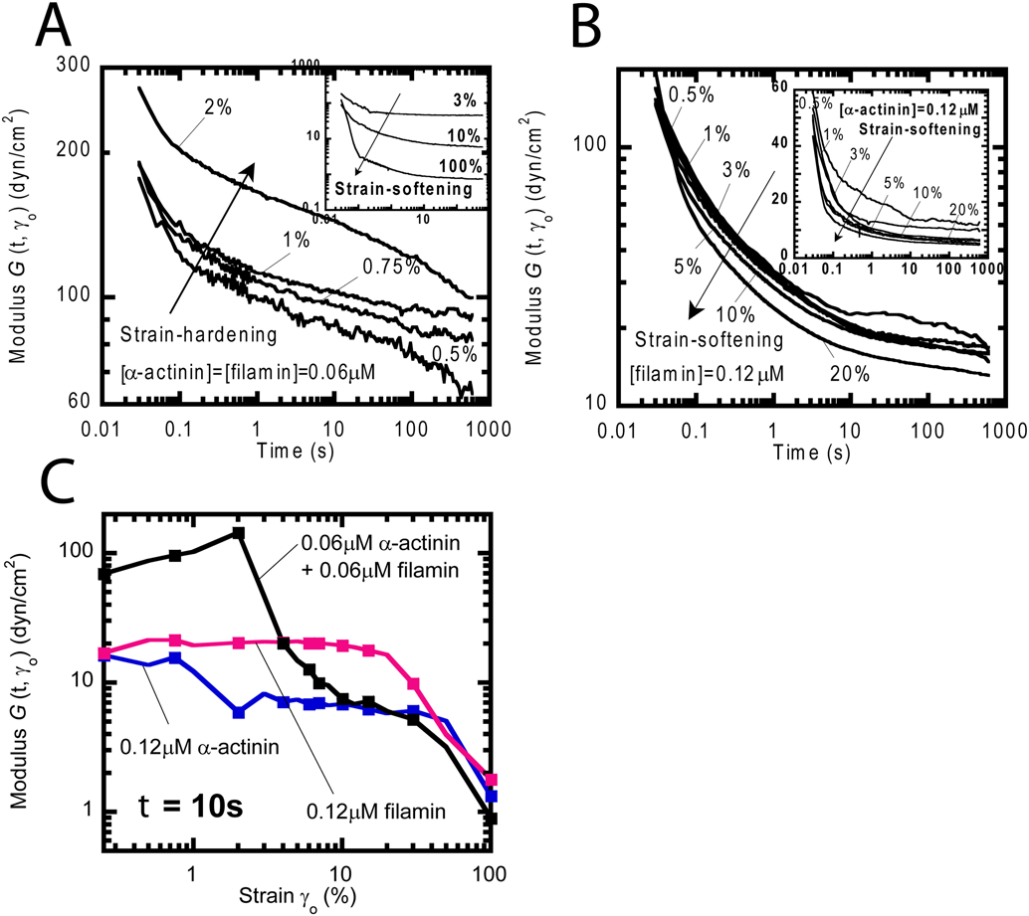
Non-linear rheology of F-actin networks in the presence of α-actinin, filamin or both. A and B. Time-dependent shear modulus *G*(t, γ_0_) of an F-actin network in the presence of (A) both α-actinin and filamin or (B) filamin alone for low shear deformation amplitude γ_0_. The modulus increases for increasing deformation amplitudes, indicating shear-induced network hardening (or stiffening). *Inset*, Time-dependent shear modulus of the same network for high deformation amplitudes. The modulus decreases for increasing deformation amplitude, indicating shear-induced network softening. C. Shear modulus of F-actin networks in the presence of α-actinin, filamin, or both as a function of deformation amplitude. The modulus is estimated at a time of 1 second.

So far we have investigated the mechanical response of F-actin networks subjected to deformations of amplitude small enough that these perturbations do not structurally change the organization of the networks. In this regime of small deformations, the magnitude of the stress induced in the network increases linearly with the input deformation and the elastic modulus is independent of the magnitude of the deformation. Here we investigate the rheological response of actin filament networks subjected to large deformations, which describes non-linear rheology. Actin filament networks containing α-actinin and filamin were subjected to step deformations of increasing amplitude (Fig. 4, A and B). The step deformation (strain) of amplitude γ_0_ induces a stress, σ, which eventually relaxes due to the movements of the filaments in solution. The modulus, *G*(t,γ_0_) =  σ(t,γ_0_)/γ_0_, of the network is measured and a resulting yield strain, γ_c_ is calculated. γ_c_ is defined as the strain required for *G*(t,γ_0_) to be reduced by 10%.

When γ_0_<γ_c_, the induced stress σ is proportional to the applied deformation amplitude γ_0_ and the network modulus *G* is independent of γ_0_ (Fig. 4C). For γ_0_>γ_c_, the modulus steadily decreased over all time scales probed presumably due to breakage or rearrangement of filaments within the network (Fig. 4C). Solutions containing either 0.12 μM α-actinin or 0.12 μM filamin had similar profiles with the modulus reducing with applied deformation with γ_c_ of ∼5% and 8%, respectively (Fig. 4C). On the other hand, solutions with both α-actinin and filamin present, exhibited strain-hardening, a phenomenon in which the modulus, *G*(t,γ_0_), increases with increasing applied shear deformation (Fig. 4, A and C). The modulus increased from 70 to 150 dyn/cm^2^ and then rapidly declined with a γ_c_ of ∼2%. Although networks containing both α-actinin and filamin display higher elasticity and increased resilience than networks formed by individual auxiliary proteins, they also break at a lower shear deformation (Fig. 4C).

## Discussion

Filamin and α-actinin are vital to structural functions of cells [6]; they are localized to both the leading edge lamelipodia as well as the trailing stress fibers [5], [7]. At the leading edge, in addition to fast remodeling of F-actin, both filamin and α-actinin can crosslink actin filaments to provide sufficient stiffness required for cells to protrude; while in stress fibers of adherent cells, where significant mechanical strength is required, filamin and α-actinin bundle actin filaments. Previous rheological studies showed that they crosslink actin filaments up to the threshold concentration and then begin to bundle these filaments [15], [17]. While these proteins can individually perform these functions, they may not be present is sufficient molar quantities to provide the mechanical integrity required by the cell.

Despite a relatively similar molecular architecture–two actin-binding sites separated by a long flexible molecular arm– and similar affinity for F-actin crosslinking/bundling proteins filamin and α-actinin modulate the mechanical properties of F-actin networks differently. F-actin networks in the presence of low concentrations of filamin form mostly orthogonal structures (i.e. no filament bundles are present) and soften (reduces their elastic modulus) under shear stresses of increasing magnitude. F-actin/filamin networks display a relatively high phase angle, similar to that shown by F-actin network without crosslinking proteins [17]. Moreover, filaments in F-actin/filamin networks with low filamin concentrations display a mobility similar to filaments in F-actin networks. However, past a critical crosslinking-to-bundling transition concentration, F-actin/filamin networks strain-harden under shear stresses and display a small phase angle, i.e. these networks feature a much more solid-like rheological character than F-actin networks and F-actin/filamin networks at low filamin concentrations [17]. In striking contrast, F-actin networks containing α-actinin strain-harden under increasing shear stresses over a wide range of α-actinin concentrations. They also display a phase angle that decreases steadily for increasing α-actinin concentration, through the crosslinking-to-bundling transition concentration [18]. Our results suggest that combining these structurally similar, but functionally different F-actin crosslinking proteins create a novel hybrid mechanical behavior.

Our results show that actin filaments polymerized in the presence of both filamin and α-actinin form a network that is stiffer than networks formed by either protein. At a molar concentration below the bundling threshold (approximately 1∶200 and 1∶150 for filamin and actinin, respectively) for either protein [15], [17], actin filaments form loose, orthogonal networks. However when both proteins are present even at a combined concentration less than their bundling threshold, they form F-actin networks that are less labile and exhibit a network strain-hardening under large forces similar to networks formed in the presence of bundling proteins such as fascin [17], [19], [20], [24]. Above its critical concentration, filamin bundles actin filaments form networks that are stiffer than networks of mixed auxiliary proteins below their threshold concentration.

Our results complement our previous studies that showed that auxiliary proteins synergistically enhance the mechanical properties of F-actin networks to promote complex cellular functions during cell adhesion, polarization, motility and division [21], [22], [25]. Earlier studies suggested that the functions of cytoskeleton regulatory proteins may be redundant or not essential; evidence of the synergistic mechanical effect of combining these proteins presented here infers that these proteins function cooperatively to provide the cell with the necessary optimal mechanical integrity. Since these two cytoskeleton proteins are localized in stress fibers of adherent cells and to the lamellipodium of protruding cells [5], it is suggestive that α-actinin and filamin do not have redundant mechanical functions.

## Materials and Methods

### Purification of the proteins

Unless specified, all reagents were obtained from Sigma. Actin was prepared from chicken breast [22]. Mg^2+^-actin filaments were generated by adding 0.1 volume of 10-X KMEI (500 mM KCl, 10 mM MgCl_2_, 10 mM EGTA, and 100 mM imidazole, pH 7) polymerizing salt to 0.9 volume of G-actin in buffer G (0.2 mM ATP, 0.5 mM dithiothreitol, 0.2 mM CaCl_2_, 1mM sodium azide, and 2 mM Tris-HCl, pH 8). Filamin and α-actinin were purified from chicken gizzard as described [26], [27].

### Quantitative rheology

The mechanical properties of actin filament networks in the presence and absence of α-actinin and filamin were measured using a strain-controlled rheometer (ARES-100 TA Instrument, Piscataway, NJ) [28], [29]. A rheometer consists of a 50-mm diameter cone and plate that form a space in which the specimen is placed. The deadtime for specimen loading in the rheometer is 30 s. The bottom plate applies oscillatory shear deformations of controlled amplitude and frequency and the induced stress is measured. The in-phase and out-of-phase components of the stress divided by the amplitude of the input deformation (1%), i.e. the elastic modulus (or elasticity), *G'*, and the viscous modulus, *G”*, as well as the phase angle, δ = tan^−1^(*G”*/*G'*) are computed. To measure the frequency-dependent elastic and viscous moduli of the actin filament networks, *G'*(ω) and *G”*(ω), oscillatory deformations of 1% shear deformation and frequency between 0.01 and 100 rad/s are applied, respectively. Finally, step deformations of amplitude between 0.1% and 100% are applied to measure compute the shear modulus *G*(t, γ_0_) as a function of time after shear application and deformation amplitude γ_0_ and compute the mechanical resilience of the networks.

### Statistics

Statistical analysis was performed and mean values and standard error of measurement (SEM) were calculated and plotted using Graphpad Prism (Graphpad Software, San Diego, CA). Two-tailed unpaired *t* tests were conducted to determine significance of differences in elastic modulus, phase angle, and exponent a. Significance was indicated using the standard Michelin Guide scale (*** for *P*<0.001, ** for *P*<0.01, and * for *P*<0.05).
